# The Role of Exosomal MicroRNAs in the Tumor Microenvironment of Breast Cancer

**DOI:** 10.3390/ijms20163884

**Published:** 2019-08-09

**Authors:** Qingqing Liu, Fu Peng, Jianping Chen

**Affiliations:** 1 School of Chinese Medicine, Li Ka Shing Faculty of Medicine, The University of Hong Kong, Pokfulam 999077, Hong Kong, China; 2 HKU Shenzhen Institute of Research and Innovation, Shenzhen 518057, China; 3 West China School of Pharmacy, Sichuan University, Chengdu 610041, China

**Keywords:** breast cancer, tumor microenvironment, exosomes, microRNA, biomarker

## Abstract

Breast cancer, ranking first among women’s cancers worldwide, develops from the breast tissue. Study of the breast tissue is, therefore of great significance to the diagnosis and treatment of breast cancer. Exosomes, acting as an effective communicator between cells, are in the ascendant in recent years. One of the most important cargoes contained in the exosomes is microRNAs, belonging to the non-coding RNA family. When the exosomal microRNAs are absorbed into the intracellular location, most of the microRNAs will act as tumor promoters or suppressors by inhibiting the translation process of the target mRNA, thus affecting the behavior of other stromal cells in the tumor microenvironment. At present, growing research focuses on the different types of donor cell sources, their contribution to cancer, miRNA profiling, their biomarker potential, etc. This review aims to state the function of diverse miRNAs in exosomes medicated cell–cell communication and the potency of some specific enriched miRNAs as molecular markers in clinical trials. We also describe the mechanism of anti-cancer compounds through exosomes and the exploration of artificially engineered techniques that lead miRNA-inhibitors into exosomes for therapeutic use.

## 1. Introduction

### 1.1. Breast Cancer

Breast cancer is one of the most common female malignant tumors and threatens the physical and psychological health of women around the world. About 1.3 million cases of mammary cancer worldwide are diagnosed and 450,000 people’s lives are being taken every year [1]. Moreover, it was reported by the American Cancer Society that breast cancer ranked first in the incidence rate among American women′s cancer between 1975 and 2014 [2], ranking second in mortality (after lung cancer) [3]. According to the latest report, there were approximately 268,670 new breast cancer patients in 2018 in the United States, which resulted in 40,000 deaths [2].

Breast carcinoma is a complex disease of morphological and molecular heterogeneity, characterized by three morphological grades and over four different molecular subtypes (at the gene expression level) [4]. According to the consensus reached at the St. Gallen International Expert Conference of breast cancer in 2015 and 2017 [5,6], breast cancer was clinically classified into four major subtypes: triple negative, hormone receptor (HR)-negative and human epidermal growth factor receptor 2 (*HER2*)-positive, HR-positive and *HER2*-positive, HR-positive and *HER2*-negative (Table 1). For different types of breast tumors, there are great differences in the treatment options.

### 1.2. Tumor Microenvironment (TME)

As known to us all, the constant growth of tumor metastasis is responsible for most cancer deaths [9]. Since Paget first proposed the famous ‘seed and soil’ hypothesis (1989), the relationship between the microenvironment and the tumor has caused widespread concern that tumor metastasis was not an accidental event, it happened only when those cancer cells with potential to metastasize (the ‘seed’) were compatible and familiar with proper organ microenvironment (the ‘soil’) [9,10,11]. The TME often refers to an area that is close to the existence of the solid tumor. Apart from breast cancer cells, the TME also contains plenty of other different types of cells including vascular endothelial cells (VECs), cancer-associated fibroblasts (CAFs), immune cells like tumor-associated macrophages (TAMs), myeloid-derived suppressor cell (MDSCs), T lymphocytes, B lymphocytes, as well as myoepithelial cells, adipocytes, etc. Moreover, some non-cellular components are also involved, covering the extracellular matrix (ECM), exosomes, soluble cytokines or signaling molecules [12,13]. It is worth noting that the physical characteristics of the tumor microenvironment are also different from normal tissues, such as hypoxia, acidity, high interstitial fluid pressure [13,14].

Cancer-associated fibroblasts (CAFs), which are considered as ‘activated fibroblasts’, constitute a major intracellular component of tumor stroma in the microenvironment [15]. CAFs can be derived from quiescent fibroblasts with altered phenotype and effects [16], epithelial cells through the epithelial-mesenchymal transition (EMT) [15,16,17], endothelial cells through the endothelial- mesenchymal transition (EndMT) [17,18], bone marrow-derived cells [19,20], and so on [18]. Through the secretion of different types of cytokines and growth factors, CAFs can have interactions with cancer cells, inflammatory cells, and other various cells and affect the occurrence and progression of tumors. For example, CAFs can secrete stromal-cell-derived factor 1 (SDF-1/CXCL12) [21], vascular endothelial growth factor (VEGF) [22], platelet-derived growth factor (PDGF) [18], fibroblast growth factor (FGF) [23], etc., to induce angiogenesis and promote tumor cells’ proliferation; degrade and remodel ECM by producing the members of matrix metalloproteinase family (MMPs) [24], resulting in the decrease of the ability of cell adhesion and contribute to metastasis. There are certain effects on the local immunity of tumors [16] by secreting interleukin-6 (IL-6), IL-10, IL-8, C-X-C motif chemokine ligand 9 (CXCL9), CXCL10, etc.

As described by Kalluri et al. [15], tumors can also be seen as a wound, accompanying inflammatory reactions. Different immune cells in the tumor microenvironment have different effects, thus creating a balance between carcinogenesis and tumor suppressor. Tumor-associated macrophages (TAMs) belong to bone marrow-derived cells with important roles in innate and adaptive immunity [25]. They are very abundant and highly infiltrating in the tumor microenvironment, and the richer density the macrophages, the worse the prognosis of patients [26]. TAMs can be derived from the following types of cells: blood monocytes, blood monocyte-related myeloid-derived suppressor cells, tissue-resident macrophages [27]. They can be recruited to tumor sites by cytokines (colony-stimulating factor-1(CSF1), chemokine (C–C motif) ligand 2 (CCL2), CCL5, etc.), and differentiate into TAMs [27]. Generally speaking, there are two subtypes of TAMs— classically (M1)- and alternatively-activated (M2) macrophages [12]. M1 macrophages have antineoplastic effect with the function of secreting tumor necrosis factor-α (TNF-α), IL-23, IL-12, etc., while M2 phenotype will express IL-10, CCL20, transforming growth factor-β (TGF-β), etc., to promote tumor [28]. In addition, TAMs can excrete cytokines such as epidermal growth factor (EGF), PDGF, VEGF, CCL2, CXCL8 to promote angiogenesis [29]; participate in CSF1 (secreted from breast cancer cell) and EGF (contributed by activated macrophages) feedback loop to cause metastasis [29]; and accumulate in hypoxic area [26].

In brief, it must be emphasized that tumor microenvironment is critical for tumor behaviors: occurrence, progression, invasion and metastasis, prognosis and drug resistance [29,30].

### 1.3. Exosomes

Exosomes, a branch of extracellular vesicles (EVs), are encased in cell membranes made up of lipid bilayers with a diameter of 30–100 nm and cup-shape appearance [31,32]. Almost all eukaryotic cells can secrete exosomes, including tumor cells and normal stromal cells, but it is reported that cancer cells express more exosomes than normal proliferation cells [32,33].

The biogenesis of exosomes [34,35,36] mainly consists of the following steps: (1) cell endocytosis: inward depression to form early endosome and enclose some intracytoplasmic contents (2) gradually mature to form late endosomes, also known as intracellular multivesicular bodies (MVBs); and (3) the MVBs will be degraded by lysosome or recycled by fusing with the plasma membrane and releasing the intraluminal vesicles (ILVs), namely exosomes. Interestingly, different cells may select different contents into exosomes, but the selection mechanisms need to be further explored [34]. Accordingly, exosome has various surface receptors, including lipids (phosphatidylserine), sialoadhesin (CD169), tetraspanins (CD9, CD63, CD81, etc.), antigen presentation (MHC I, MHC II), and adhesion molecules (integrins, lactadherin, etc.) [36]. There are also several main mechanisms for the process of recipient cells’ uptake [37]: (1) the T cell receptor- major histocompatibility complex (MHC) interaction; (2) fusion with membrane of recipient cells; (3) cell phagocytosis; and (4) adhesion molecules interaction.

Recently, exosomes have come under increasing interest from researchers, mainly because they have been found to wrap many biomolecules, such as DNAs, mRNAs, non-coding gene family (microRNA, lncRNA), proteins, and lipids [38]. Carrying these active biomolecules, exosomes have also been found to circulate in body fluids, such as blood (plasma or serum), urine, feces, breast milk and saliva, so that exosomes are able to transmit intercellular regulatory information to the both surrounding and distant sites, which indicates the potential to be non-invasive biomarkers [39]. Increasing articles of exosomes reported extensive involvement in physiological and pathological processes, such as placental physiology [40], angiogenesis and increased heart function [41], endometrial-embryo interactions [42], and pituitary tumors [43]. Here, we mainly focus on the role of exosomes in breast cancer.

### 1.4. MicroRNAs

In 1993, Lee et al. reported the first discovery of miRNA that gene lin-4 encoded small RNA molecules to regulate the expression of protein lin-14 in *Caenorhabditis elegans* [44]. MicroRNAs (miRNAs) are small RNA molecules with single chains that do not encode proteins. They usually have a length of about 19 and 25 nucleotides, most of which negatively regulate the post-transcriptional level of target messenger ribonucleic acids (mRNAs) by binding to the 3′-untranslated region (UTR) [45]. Three possible mechanisms may be involved in the process: repression of translation initiation, post-initiation inhibition and target mRNA destabilization, resulting in the degradation of mRNA or translation inhibition [46].

In recent years, numerous studies have shown that varieties of microRNAs function in direct or indirect interactions between breast cancer cells and components of TME [47,48]. For example, miR-373 and miR-520c can raise the invasion and metastasis of tumor by inhibiting metastasis-related gene CD44 [49]. Moreover, miRNAs can be circulated in body fluid and the expression of miRNAs in peripheral blood may be used as a biological marker for the differential diagnosis and prognosis of breast tumors [7,39]. Exosomal miRNA profiling analyses were reported and compared, such as between drug-resistant and non-drug-resistant cells [50,51], normal mammary epithelial cells (MCF-10A) and MCF-7 [52], ER-positive (MCF-7) and triple negative (MDA-MB-231) cancer cell lines [53], HER2-positive and triple negative cancer patients [54], from canine mammary cells [55]. Liu et al. [56] integrated small RNA sequencing information of 17 diseases and built up an open EVmiRNA database for public searching of miRNA profiles.

This review mainly focuses on the bioactivity and underlying mechanism of exosomal microRNAs in the change of tumor microenvironment during the initiation and development of breast cancer.

## 2. The Role of miRNAs in Exosomes in the Intercellular Crosstalk

### 2.1. Exosomes from Cancer Cells Can Provide MicroRNAs to Modify the Stromal Cells in the Tumor Microenvironment for Their Own Advantage (Table 2)

The synthesis of microRNAs may occur in extracellular microvesicles. Melo et al. [57] reported that exosomes derived from mammary tumor cells (MDA-MB-231, MCF-7, 4T1) can specifically aggregate pre-miRNAs, as well as other proteins of the RISC complex, and then generate the mature miRNAs (up-regulated: miR-10a, -10b, -21, -27a, -155, -373) inside. When non-tumorigenic mammary epithelial cells (MCF-10A) and exosomes of tumor cells (MDA-MB-231) were co-cultured, the cancerization of normal cells was promoted, and Dicer enzyme (an important instrumental enzyme in the formation of mature miRNAs) could be seen as a controlling factor of this process. When MCF-10A was co-injected with exosomes derived from serum of breast cancer patients or healthy controls into nude mice, the former could form tumors with higher level of Dicer while the latter could not. Furthermore, exosomal miR-210 from tumor cells could transfer to endothelial cells to promote angiogenesis and metastasis and the secretion process of exosomes was also proved to be dependent on neutral sphingomyelinase 2 (nSMase2) enzyme (regulate ceramide biogenesis) [58,59]. Singh et al. [59] proved that nSMase2 or ceramide could promote the level of exosomal miR-10b, resulting in enhanced invasion ability of non-malignant cells by inhibiting the expression of homeobox D10 (HOXD10) [60] and KLF4. Other miRNAs such as miR-1246 [61] were also found to be secreted from breast cancer cells and change the behaviors of normal epithelial cells (Table 2).

Some highly metastatic/drug-resistant tumor cells can affect other tumor cell lines with low metastatic/non-drug resistance by secreting miRNAs in the exosomes, which is more convenient for their excessive growth. Different chemotherapeutic drugs-resistant MCF-7 cell lines were reported to secrete different miRNAs such as miR-222 [62,63], -23a [64], -100 [65], and -149 [66], etc., to non-drug-resistant cells to improve their resistance, leading to treatment failure (Table 2). In addition, some in vitro experiments also showed that after incubating purified exosomes from the metastatic cell line MDA-MB-231, the mobility and anchorage-independent ability (metastatic behaviors) of low metastatic MCF-7 cells were all increased and the underlying mechanism was related to exosomal miR-9 and miR-155 [67,68]. Genetically manipulated cells can also increase the level of specific microRNAs in exosomes and be absorbed by the original cell lines [69,70], which prompts a therapeutic direction.

**Figure 1:**
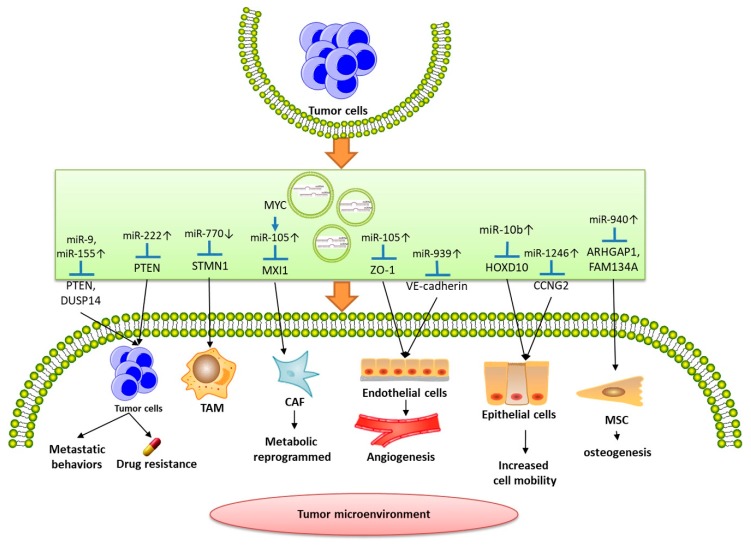
Tumor cells can secrete exosomes which contain diverse microRNAs to modify low-metastatic or non-resistant cancer cells and the stromal cells in the tumor microenvironment such as tumor-associated macrophages (TAMs), cancer-associated fibroblasts (CAFs), endothelial and epithelial cells, mesenchymal cells (MSCs) for their own advantage.

Not only breast-related cells, but other stromal cells in the microenvironment can also uptake the external exosomes derived from the tumor cells, and then the contained miRNAs will be transported into them and affect their behavior, providing a favorable environment for the growth of the tumor (Figure 1). Exosome-mediated transfer of miR-105 [71] and miR-939 [72] could increase the permeability and destroy the integrity of vascular endothelial cells, leading to enhanced metastasis by targeting the zonula occludens 1 (ZO-1) gene and VE-cadherin, respectively. From data of clinical trials, exosomal miR-105 was proved to be associated with the incidence of metastasis [71]. Yan’s study [73] also demonstrated that the level of miR-105 increase in the exosomes secreted from breast cancer cells was induced by oncogene MYC, and then transferred to CAFs. They also showed that miR-105 targeted at MAX-interacting protein 1 (MXI1) to activate MYC signaling and reprogram CAFs. Interestingly, whether under the circumstances of efficient nutrition or not, reprogrammed CAFs would ultimately promote tumor growth by leading to different metabolic pathways (glucose and glutamine metabolism with sufficient or metabolic waste decomposition, such as lactic acid and ammonium) and regulating the components of TME to provide energy to fuel cancer cells [73], which was similar to exosomal miR-122 transferred to lung fibroblasts and brain astrocytes [74]. In a recent study, after overexpressing miR-940 in breast cancer cells, treatment of human mesenchymal stem cells (MSCs) with exosomes contained culture medium- was shown to promote osteogenesis in vitro by acting on Rho GTPase-activating protein 1 (ARHGAP1) and family with sequence similarity 134, member A (FAM134A) [75]. The well-designed in vivo experiment also demonstrated that exosomes (labelled with CD63 (exosomes’ marker) fused red fluorescent) were derived from miR-940 overexpressed cancer cells and absorbed by host cells (labelled with GFP) under confocal microscopy, which caused osteoblastic lesions by implanting cancer cells onto the calvarial bones/tibial sites of immunodeficient mice [75]. The growing expression of miR-770 (acting as a tumor suppressor, direct targets at STMN1) from TNBC cells to tumor-associated macrophages (TAMs) through exosome delivery modulated more differentiation to M1 phenotype instead of M2 phenotype and inhibited drug-resistance [76].

Cancer-produced exosomes may also be regulated by the physical conditions of microenvironment like hypoxia, an unignored feature of solid tumors that motivates tumor deterioration [77]. By using a miR-210 specific reporter system, elevated miR-210 was visually proved to be transferred from hypoxic cancer cells to proximal endothelial cells via exosomes both in vitro and in vivo, which may be interceded by hypoxia-inducible factor-1α (HIF-1α) and restrain the expression of vascular remodeling related genes, like Ephrin A3 and PTP1B, to support angiogenesis and tumor growth [78,79].

### 2.2. Exosomes from Stromal Cells Can Transfer miRNAs to Cancer Cells and Contribute to Cancer Progression (Table 3)

**Figure 2:**
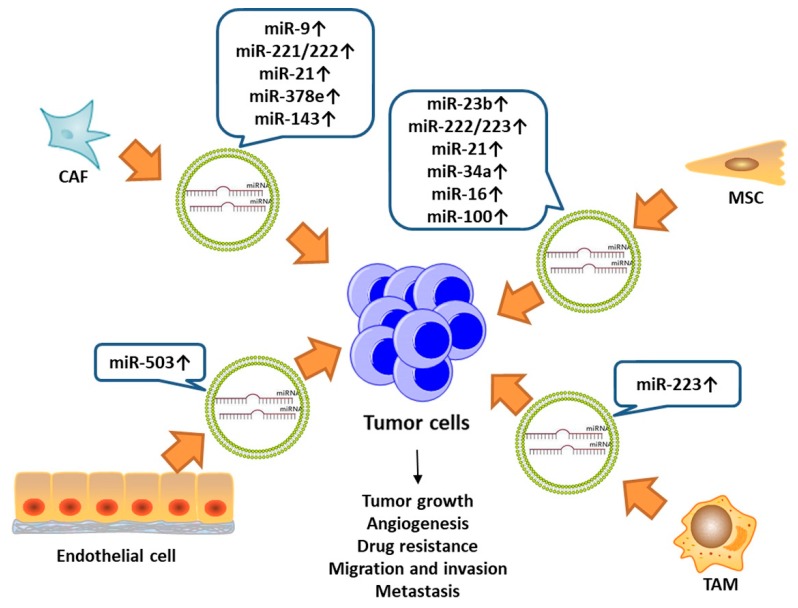
Exosomal microRNAs from stromal cells could enter and function in cancer cells and contribute to cancer progression including tumor growth, angiogenesis, metastasis, etc.

In turn, as the reports accumulated, stromal cells can also express exosomes to function in cancer cells (Figure 2). For instance, miR-9 can be secreted in exosomes by both breast cancer cells and CAFs [80]. When miR-9 is transferred from tumor cells, it can enhance the transformation of normal fibroblasts (NFs) to CAFs and its migration and invasion abilities. Conversely, CAFs can secrete miR-9 to tumor cells and NFs to promote tumor growth [80]. Dioufa et al. [81] observed that the tumor-derived exosomes helped to transfer the tumor cells to the liver and then remain in the dormancy state in the early stage of metastasis, which was characterized in slow proliferation, insensitivity to chemotherapy and difficulty in discovery, resulting in recurrence and poor prognosis [82]. However, exosomes from hepatic niche cells led to an increased mesenchymal to epithelial reverting transition (MErT) of cancer cells, which was explained as the adaptation process of tumor cells in heterogeneous organs [81]. Uen et al. [83] demonstrated that miR-122-5p, which was found in the human hepatoma cells’ exosomes, would target at syndecan-1 (SDC-1) and promote breast cancer cell mobility.

A relatively higher level of miR-155 was detected in exosomes of breast cancer stem cells (CSC) and chemo-resistant cancer cells. When sensitive cancer cells were co-cultured with resistant cells’ exosomes, the migration ability and chemoresistance was promoted [84]. Exosomal miR-23b was reported to transit from bone marrow mesenchymal stem cells (BM-MSCs) to metastatic breast cancer cells which were homing to bone marrow (BM2), and the BM2 cells could be induced to enter into dormancy by inhibiting myristoylated alanine-rich C kinase substrate (MARCKS) [82]. Similarly, miR-222/223 [85], miR-127 and miR-197 [86], miR-21 and miR-34a [87], miR-126a [88] in exosomes from BM-derived cells also supported tumor growth in different ways such as drug resistance, dormancy, metastasis, and angiogenesis (Table 3).

Interestingly, CAFs could secrete exosomes encapsulating with miR-221/222 [89], miR-21, -378e, -143 [90] to enhance the development of cancer cells to a more aggressive phenotype with increased stemness, EMT ability, etc. Additionally, exosomes from interleukin-4 (IL-4) activated TAMs, upregulated the expression of miR-223 in breast cancer cells with co-culture systems, which further promoted the invasiveness of cancer cells by disrupting the Mef2c-β-catenin pathway [91].

However, there are some reports that exosomal anti-cancer miRNAs would also be secreted by stromal cells to fight with the malignancies (Table 3). MSC-derived exosomes shuttled miR-16 [92] and miR-100 (inhibiting the mTOR/HIF-1α/VEGF pathway) [93] to the nearby cancer cells and decreased their VEGF expression, subsequently resulting in inhibition of the endothelial cells’ vascular behavior. After chemotherapy, the upregulation of miR-503 via exosomes in endothelial cells reduced cancer cells’ proliferation and invasion through targeting cyclin D2 (CCDN2) and CCDN3 [94].

### 2.3. Circulating MicroRNAs in Exosomes Imply Their Potential Biomarker Value

Some circulating microRNAs contained in exosomes can potentially be regarded as predictive, diagnostic and prognostic biomarkers for breast cancer (Table 4). The advantages of exo-miRs over miRs are obvious: (1) Exo-miRs act as cell-cell communicator and circulate in the body fluids, such as peripheral blood andbreast milk. Thus, the collection method is non-invasive, or simply a common method like blood collection. When it comes to miRs, it is not easy to get tumor tissues and pathological diagnosis is still the gold standard. (2) It has also been reported that the expression level of some special exo-miRs have shown significant differences in early stages of cancer, even during dormancy period. In this case, miRs in tumor tissues cannot be detected. (3) Post-operative monitoring is even more important because recurrence and metastasis are still the greatest cause of breast cancer death. Additionally, exo-miRs can be easily detected and constantly monitored.

Plasma- and serum-derived miR-106a-5p and miR-20b-5p, belonging to miR-106a-363 cluster on chromosome X, showed an increasing trend in tissues and exosomes, indicating their role as potential diagnostic biomarkers [95]. In addition, a combination of multiple markers may also be a possible way to indicate the presence of breast cancer. For example, exosomal miR-12 and miR-1246 were proved to be a potential combined indicator [96]. Cell-free miR-101 and miR-373 in blood serum exhibited significant differences between malignant and benign tumors, and it is also worth noting that the higher content of exosomal miR-373 was associated with malignant breast cancer like TNBC [97]. Gao et al. [98] also reported that high level of miR-155, which may be derived from blood cells exosomes, was found in the early stage in cancer patients. Additionally, miR-130a-3p (tumor suppressor) [99], miR-16, miR-30b and miR-93 [100], miR-200c and miR-141 [101], and miR-223-3p [102] in exosomes also exhibited valuable functions to classify cancer stages (Table 4).

Recurrence often happens in breast cancer patients even after a mastectomy, which is totally different from patients never being treated, so the development of molecular markers for recurrence and treatment monitoring is very essential. High level of exosomal miR-21, -105, and miR-222 in serum samples [103] could possibly become a complementary tool for prognostic and monitoring use clinically because the level of miR-21 was proved to link with tumor size and Ki-67 expression during the treatment. The miRNA profile was detected in clinical patients with or without recurrence, and the level of miR-340-5p, miR-17-5p, miR-130a-3p, and miR-93-5p exhibited close relation with recurrence rate in their logistic regression analysis [104].

When the sample (e.g., plasma, serum) are obtained, the usual method is to use ultracentrifugation or commercial kits to extract and purify exosomes, and then use the transmission electron microscope (TEM) to detect their diameter size (30–100 nm), western blot (WB) to determine surface biomarkers (such as CD63, CD81, CD9), and other methods to confirm that the isolated EVs are exosomes, and to preserve in low temperature afterwards. When testing microRNAs, experimental methods such as RNA sequencing or qRT-PCR will be used. Exosomes uptake can be observed by PKH67 staining or confocal microscopy detection of fluorescence. Furthermore, since miRNAs can easily get degraded with time, different in situ and quantitative methods were also explored in order to detect the content of exosomal miRNAs in a faster and better manner. A direct method without RNA isolation or purification procedures, which was also evaluated in the terms of specificity, accuracy and efficiency, was used to prove miR-106a in plasma as a potential diagnosis biomarker [105]. Zhai et al. [106] synthesized an in situ probe which can quantitatively evaluate the level of miR-1246 in plasma exosomes with high degree of sensitivity and specificity, which is a promising translation for clinical use. Likewise, other reported methods for detecting exosomal miRNAs are mainly based on biosensors [107,108]; fluorescent probes [109,110]; DNA enzyme probes [111]; green fluorescent protein (GFP) tag technology [112]; weight-dependent molecular sieves [113], etc.

It is necessary to develop more effective, specific, sensitive biomarkers and detection methods to accelerate detection speed and improve patients’ compliance. In the future, this kind of reagent will make it possible for a single drop of blood to verdict breast cancer, different from subtypes and treatment response monitoring.

## 3. Therapeutic Method Targeting at the Exosomal microRNAs

The important role of exosomal miRNAs in cancer progress cannot be ignored, so it is also significant (1) to develop new drugs targeting at vital miRNAs and (2) to achieve a novel treatment of the exosomes with therapeutic microRNAs.

The mechanisms of some clinical anticancer drugs from natural herbs or synthetic sources are reported to be partly dependent on miRNAs as exosomal cargoes (Table 5). D Rhamnose β-hederin (DRβ-H), a triterpenoid saponin, could reduce the growth and apoptosis of cancer cells by inhibiting the secretion process of exosomes. Subsequently, some specifically encapsulated miRNAs, including miR-130a and miR-425, were also downregulated [114]. Shikonin (SK) could also suppress exosome release accompanying with reduced miR-128 [115] and restrain the cancer-promoting effects of preadipocytes through disturbing miR-140/SOX9 signaling [116]. Epigallocatechin gallate (EGCG) is being pointed out to express anti-cancer activity by inhibiting the macrophages infiltration in TME through upregulating miR-16 in exosomes [117], and chemo-susceptibility could be elevated by β-elemene through affecting the expression of resistance-related genes such as miR-34a, miR-452, Pgp, and PTEN [118]. Hannafon et al. [119] reported that after docosahexaenoic acid (DHA) administration, exosomes secreted from breast cancer cells mediated the increase of miR-23b and miR-320b in recipient endothelial cells and decreased the expression of target genes and corresponding proteins (PLAU, AMOTL1, NRP1, ETS2, respectively), thus inhibiting their tube formation and angiogenesis capability.

Increasing evidence indicated that exosomes are important carriers and they probably can be manipulated to deliver tumor suppressor-miRNA or oncomiR-inhibitors to express their potential therapeutic effects (Table 5). As described, exosomal let-7a from donor cells was specifically delivered to epidermal growth factor receptor (EGFR)-expressing tumor sites for therapeutic use [120]. Recently, O’Brien et al. [121] genetically modified MSCs by lentiviral transduction to enrich miR-379 in derivative extracellular vesicle (EV), which showed significant anti-cancer activity in vivo by direct delivery. Meanwhile, tumor-derived exosomes could also be engineered to overexpress miR-155, -142, and let-7i by electroporation to mature dendritic cells and to trigger the immunity process [122], to load siRNAs or miRNAs by sonication to knockdown oncogene like HER2 [123]. Co-transfection of antagomiR-222/223 into MSCs reversed tumor dormancy and drug resistance [85]. Gold nanoparticle (AuNP)-based gene silencing technology could also load anti-gene/miRNA oligonucleotides [124], and locked nucleic acid (LNA)-modified BM-MSCs could secrete anti-miR-142-3p oligonucleotides in exosomes and exert inhibitory effects in vitro and in vivo [125].

## 4. Conclusions and Future Perspectives

In the tumor microenvironment of breast cancer, the powerful regulatory effect of the exosomal miRNA is obvious and true. Tumor cells, especially with the phenotype of malignancy and drug-resistance, would secrete exosomes containing specific miRNAs to non-drug-resistant cancer cells, and even to normal stromal cells. Conversely, macrophages, fibroblasts, and other stromal cells could also transmit the exosomes to the cancer cells, which encouraged the erosion of cancer cells.

Although some progress has been made in this field, and some advanced understandings have arrived, we still face difficulties. For example, there is only little research on the response of exosomes to the changes of physical conditions during the carcinogenesis such as acidity and hypoxia in TME. Additionally, no systematic and accepted method has been found in the separation of the exosomes from plasma or serum yet, which may result in a non-reliable outcome. It is recognized that the clinical use of molecular markers is very demanding. The conversion of exosomal miRNA in the peripheral blood, which is clearly enriched and differentiated in basic scientific research, into a clinical biomarker for diagnosis and detection requires more detailed screening and more support of clinical data. A greater effort needs to be taken to exploit the deeper potential of miRNAs in exosomes, such as revealing their mechanisms in depth, the modification of exosomes, and the development of new clinical treatments.

## Figures and Tables

**Table 1:** Subtypes of breast tumor (summarized from St. Gallen conference of 2015 and 2017).

Subtypes	Classification	*HER2*	HR
Triple negative	TNBC ^1^	(−)	ER ^2^ (−), PgR ^3^ (−)
HR (−) and *HER2* (+)	*HER2*-positive	(+)	ER (−), PgR (−)
HR (+) and *HER2* (+)		(+)	ER and/or PgR (+)
HR (+) and *HER2* (−)	Luminal-A like	(−)	ER and/or PgR (+); Multi-parameter molecular marker ‘good’ if available; High ER/PR; clearly low Ki-67 (low proliferation [7]); low grade (well-differentiated [8])
	Intermediate	(−)	Multi-parameter molecular marker ‘intermediate’ if available.
	Luminal-B like	(−)	ER and/or PgR (+); Multi-parameter molecular marker ‘bad’ if available; Lower ER/PR; clearly high Ki-67 (high proliferation [7]); histological grade 3 (poorly differentiated [8])

^1^ TNBC, triple negative breast cancer; ^2^ ER, estrogen receptor; ^3^ PgR, progesterone receptor.

**Table 2:** Evidence supporting a role for exosomal microRNAs in cancer cell-cell communication (from cancer cells).

Stimulant	Cargo-microRNAs	Donor Cells	Recipient Cells	Gene Targets	Biological Activities	Major Findings	Refs
	miR-10a, 10b, 21, 27a, 155, 373 (↑)	Cancer cell (MDA-MB-231, MCF-7, 67NR, 4T1)	Epithelial cells (MCF-10A, NMuMG)	HOXD10 (miR-10b), PTEN (miR-21)	Tumorigenesis (in a Dicer-dependent manner)	Pre-miRNAs, Dicer (CD43-mediated accumulation), AGO2, and TRBP are present in exosomes of cancer cells to generate mature miRNAs.	[57]
	miR-210 (↑)	Cancer cell (4T1, MCF-7, MDA-MB-231-D3H1, MDA-MB-231-D3H2LN)	Endothelial cells (HUVECs)	ephrin-A3	Angiogenesis; metastasis	nSMase2 is important to regulate exosomal miRNAs, which will transfer to endothelial cells to promote metastatic initiation efficiency.	[58]
Twist	miR-10b (↑)	Cancer cell (MDA-MB-231, MCF-7)	Epithelial cells (MCF-10A, HMLE)	HOXD10 (inhibit the expression of the pro-metastatic gene, RHOC), KLF44	Invasion	nSMase2 or ceramide promotes the exosome-mediated miR-10b secretion.	[59,60]
	miR-1246 (↑)	Cancer cell (MDA-MB-231, MCF-7)	Epithelial cells (MCF-10A, HMLE)	CCNG2 (tightly regulated through the cell cycle)	Cell proliferation; invasion; drug resistance	Exosomal miR-1246 functions in regulating breast tumor progression and has the potential for applications in miRNA-based therapeutics.	[61]
	miR-221/222 (↑)	Cancer cell [MCF-7/Tam]	Cancer cell [MCF-7/WT (tamoxifen sensitive)]	ERα, p27 (cell cycle arrest, autophagy, and angiogenesis)	Drug resistance (tamoxifen)	EV-secreted miR-221/222 serves as signaling molecules to mediate the communication of tamoxifen resistance.	[62]
	miR-222 (↑)	Cancer cell (MCF-7/Adr)	Cancer cell (MCF-7/sensitive)		Drug resistance (adriamycin)	Exosomes are effective in transmitting drug resistance and the delivery of miR-222 via exosomes may be a mechanism.	[63]
	miR-23a, 29a, 1246, 222, 452 (↑)	Cancer cell (MCF-7/Doc)	Cancer cell (MCF-7/sensitive)	Sprouty2 [regulate invasion and metastasis] (miR-23a), PTEN (miR-222), APC4 (miR-452)	Drug resistance (docetaxel)	Abundant miRNAs of Doc/exo in pathways implicated in therapy failure.	[64]
	miR-100, 222, 30a (↑)	Cancer cell (MCF-7/Doc, MCF-7/Adr)	Cancer cell (MCF-7/sensitive)	PTEN (miR-222)	Drug resistance (docetaxel, adriamycin)	The involvement of miRNAs in pathways implicated in cancer pathogenesis, membrane vesiculation, and therapy failure.	[65]
	miR-23a, 24, 149, 222 (↑)	Cancer cell (MCF-7/Adr)	Cancer cell (MCF-7/sensitive)	Sprouty2 (miR-23a), PTEN p27 (miR-24), (miR-222)	Drug resistance (adriamycin)	Adr/exo loaded miRNAs for its production, release and which were associated with Wnt signaling pathway. Adr/exo was able to increase the overall resistance and regulate gene levels.	[66]
	miR-9, miR-155 (↑)	Cancer cell (MDA-MB-231)	Cancer cells (MCF-7)	PTEN (miR-9), DUSP14 (miR-155)	Tumor growth	Exosomal miRNAs can transfer from highly metastatic cancer cells to other low metastatic cancer cells and can suppress target genes in the recipient cells.	[67,68]
	miR-182 (↑)	Cancer cell (miR-182 transfected MDA-MB-231)	Cancer cell (naïve MDA-MB-231 cells)		Tumorigenesis	MiR-182 is packaged in exosomes, detectable in exosomes from cell culture supernatant and human serum, which may be transferred between cells via a microvesicle-dependent mechanism.	[69]
	miR-134 (↓)	Cancer cell (miR-134-transfected Hs578T, a TNBC cell line; isogenic sub-clone cells)	Cancer cells (Hs578Ts(i)8 parent cells)	STAT5B (control Hsp90)	Cellular proliferation; migration and invasion; drug resistance (cisplatin, anti-Hsp90 drug)	(1) The direct transfection or EV delivery transport route of miRNA achieved different effects. (2) MiR-134 had clinical relevance in breast tumors.	[70]
	miR-105 (↑)	Cancer cell (MDA-MB-231, MCF-10A as the control group)	Endothelial cells (HMVECs)	ZO-1 (also called tight junctions protein 1, migration-related gene)	Metastasis	Exosome-mediated transfer of cancer cell-secreted miR-105 efficiently destroys tight junctions and the integrity of these natural barriers against metastasis.	[71]
	miR-939 (↑)	Cancer cell (MDA-MB-231-GFP cells)	Endothelial cells (HUVECs)	VE-cadherin (a component of adherens junction involved in vessel permeability)	Migration and invasion	MiR-939 could (1) increase HUVECs monolayer permeability; (2) favor trans-endothelial migration by the disruption of the endothelial barrier.	[72]
MYC (oncogene)	miR-105 (↑)	Cancer cell (MDA-MB-231, MCF-10A as the control group)	CAFs (patient-derived primary fibroblasts CAF265922; fetal lung fibroblast cell line WI-38; mouse embryonic fibroblast cell line NIH3T3)	MXI1	Tumor growth	Reprogrammed CAFs would ultimately promote tumor growth by leading different metabolic pathways under the circumstances of efficient or insufficient nutrition.	[73]
	miR-122 (↑)	Cancer cell (MDA-MB-361, MDA-MB-468, MDA-MB-231, MDA-MB-231-HM, SKBR3, BT4, MCF-10A as the control group)	Lung fibroblast, brain astrocytes, neurons	PKM2, GLUT1	Reprogram glucose metabolism; cancer cell proliferation; metastasis	Exosomal miR-122 inhibited the glucose uptake by niche cells and increased glucose availability to cancer cells, while inhibition of miR-122 decreases the incidence of metastasis in vivo.	[74]
	miR-940 (↑)	Cancer cell (MDA-MB-231)	Human mesenchymal stem cells (MSC, UCB408E6E7TERT-33)	ARHGAP1, FAM134A	Bone metastasis	miR-940 facilitates the osteogenic differentiation of human MSCs.	[75]
	miR-770 (↓)	Cancer cell (MDA-MB-231, MDA-MB-468)	TAMs (THP-1 cell)	STMN1	Drug resistance (doxorubicin); metastasis	miR-770 could (i) influence the polarization of macrophages which promote M1 phenotype and inhibit M2 phenotype, (ii) suppress the doxorubicin-resistance and metastasis of TNBC cells	[76]
HIF-1α	miR-210 (↑)	Cancer cell (MDA-MB-231, 4T1)	Endothelial cells (SVEC), macrophages (Raw264.7), stem cells (MBs-MSC), fibroblasts (3T3), and dendritic cells (JAWS2).	Ephrin A3, PTP1B (vascular remodeling related genes)	Angiogenesis	A miR-210 specific reporter system to realize in vitro and in vivo visualization.	[78]
HIF-1α	miR-210 (↑)	Cancer cell (MDA-MB-231, SKBR3, MCF-7)	TME			Hypoxic cancer cells may release more exosomes into their microenvironment to promote their own survival and invasion	[79]
	miR-9 (↑)	Cancer cell (MDA-MB-231, MDA-MB-468)	Normal fibroblasts (isolated from specimens belonging to patients)	mainly involved in cell motility and ECM remodeling pathways	Tumor growth; migration and invasion	(1) Enhance cell motility; (2) enhance the switch to CAF phenotype	[80]

**Table 3:** Evidence supporting a role for exosomal microRNAs in cancer cell-cell communication (from stromal cells).

Stimulant	Cargo-microRNAs	Donor Cells	Recipient Cells	Gene Targets	Biological Activities	Major Findings	Refs
	miR-9 (↑)	CAFs	Cancer cell (MDA-MB-231, MDA-MB-468); Normal Fibroblasts	E-cadherin	Migration, invasion, cell proliferation	MiR-9 was an important player in the crosstalk between cancer cells and stroma.	[80]
	miR-186, 23a, -205 (↑)	The hepatic niche (HepN)	Cancer cell (MDA-MB-231)	Regulate E-cadherin transcription and MErT induction	MErT	The normal tissue/HepN derived exosomes in enabling seeding and entry into the dormancy of the cancer cells at the metastatic site.	[81]
	miR-23b (↑)	Bone marrow mesenchymal stem cells (BM-MSC)	Cancer cell (BM2 cell, MDA-MB-231)	MARCKS (encode a protein that promotes cell cycling and motility)	Dormancy; drug resistance (docetaxel)	(1) They generated a bone marrow-metastatic human breast cancer cell line (BM2); (2) Exosomal transfer of miRNAs from the bone marrow may promote breast cancer cell dormancy in a metastatic niche.	[82]
	miR-122-5p (↑)	Human hepatoma cells (Huh-7, Hep3B)	Cancer cells (MCF-7)	syndecan-1 (SDC1)	Metastasis	Metastasis or mobility of breast cancer cells might be affected by circulating miR-122-5p and not directly correlated with the progression of breast cancer.	[83]
	miR-155 (↑)	Breast CSC; DOX-/PTX-resistant MCF-7 cell line	Cancer cell (MCF-7 cell, MDA-MB-231)	TGF-β, C/EBP-β and FOXO3a	EMT; migration; chemoresistance	Exosomes may intermediate resistance, and migration capacity to sensitive cells partly through exosome transfer of miR-155.	[84]
	miR-222/223 (↑)	MSC (naive MSC; T47D, MDA-MB-231-primed MSCs)	Cancer cell (MDA-MB-231, T47D)		Cycle quiescence; dormancy; drug resistance (carboplatin)	Breast cancer cells prime MSC to release exosomal miR-222/223, which in turn promotes quiescence in a subset of cancer cells and confers drug resistance.	[85]
	miR-127, 197, 222, 223 (↑)	BM stromal cells (prepared from BM aspirates of healthy donors)	Cancer cell (MDA-MB-231, T47D)	CXCL12 (chemokine family)	Cycle quiescence; dormancy	(1) The transfer of miRNAs from BM stroma to BC cells might play a role in the dormancy of BM metastases. (2) Gap-junction maybe another way of the transfer of miRNAs.	[86]
	miR-21, 34a (↑)	Human MSC	Cancer cell (MCF-7, osteosarcoma cell)		Cell proliferation	First comprehensive-omics based study that characterized the complex cargo of extracellular vesicles secreted by hMSCs and their role in supporting breast cancers.	[87]
IL-13	miR-126a (↑)	MDSC	Cancer cell (4T1, MDA-MB-231); IL-13^+^Th2 cell	S100A9	Lung metastasis; angiogenesis	Doxorubicin treatment led to an enhancement of IL-33 in breast cancer cells, IL-13 receptor and miR-126a in MDSCs in a positive feedback loop manner.	[88]
	miR-221/222 (↑)	CAFs	Cancer cell (MCF-7 cell line long-term conditioned for growth in estrogen depleted conditions)	ER (estrogen receptor)	ER-negative phenotype	CAF-secreted microRNAs are directly involved in ER-repression and may contribute to the MAPK-induced ER-repression in breast cancer cells.	[89]
	miR-21, -378e, -143 (↑)	CAFs; Normal fibroblasts with overexpressed miRs	Cancer cell (BT549, MDA-MB-231, T47D)		Cell growth; stemness; EMT	CAFs strongly promote the development of an aggressive breast cancer cell phenotype.	[90]
IL-4	miR-223 (↑)	TAMs (isolated from the peripheral blood and activated by adding IL-4)	Cancer cell (SKBR3, MDA-MB-231)	Mef2c (inhibit proliferation and granulocyte function)	Invasion	MiR-223 may target at the Mef2c-β-catenin pathway to mediate breast cancer cell invasion.	[91]
	miR-16 (↑)	MSC	Cancer cell (4T1); Mouse endothelial cell line (SVEC)	VEGF	Angiogenesis	MiR-16 was partially responsible for the antiangiogenic effect of MSC-derived exosomes.	[92]
	miR-100 (↑)	MSC	Cancer cell (MDA-MB-231, MCF-7, T47D); Endothelial cells (HUVECs)	mTOR	Angiogenesis	MSC-derived exosomes induce a decrease in the expression and secretion of VEGF through modulating the mTOR/HIF-1α signaling axis in breast cancer-derived cells.	[93]
	miR-503 (↑)	Endothelial cells (HUVECs)	Cancer cell (A549, HCT116, MDA-MB-231, U87)	CCND2, CCND3	Cell proliferation; invasion	Increased plasmatic miR-503 in breast cancer patients after neoadjuvant chemotherapy, which could be partly due to increased miRNA secretion	[94]

**Table 4:** Preclinical evaluation of exosomal cargo as cancer biomarkers.

Cargo	Patient Cohorts	Exosome Source (Isolation Method)	Assay Used	Outcome and Utility	Refs
miR-106a-3p, 106a-5p, 20b-5p, 92a-2-5p (plasma miRNAs); miR-106a-5p, 19b-3p, 20b-5p, 92a-3p (serum miRNAs)	400 plasma samples (from 200 BC patients and 200 healthy controls (HCs)), 406 serum samples (from 204 BC patients and 202 HCs),	plasma (from 32 BC patients and 32 HCs), serum (from 32 BC patients and 32 HCs)	qRT-PCR	Except for the expression of miR-20b-5p, the expression patterns of exosomal miRNAs were concordant between plasma and serum, indicating the potential use of exosomal miRNAs as biomarkers.	[95]
miR-21, 1246 (↑)	exosomes from the conditioned media of human breast cancer cell lines, mouse plasma of patient-derived orthotopic xenograft models (PDX), and human plasma samples from 16 patients	plasma (ultracentrifugation, ExoQuick)	next-generation small RNA sequencing; qRT-PCR	The combination of plasma exosome miR-1246 and miR-21 is a better indicator of breast cancer than their individual levels.	[96]
miR-373 (↑)	168 patients with invasive breast cancer, 19 patients with benign breast diseases and 28 healthy women	serum (ExoQuick)	RT-PCR	Serum levels of exosomal miR-373 are linked to triple-negative and more aggressive breast carcinomas.	[97]
miR-155 (↑)	259 participants, including patients with breast cancer or benign breast tumors, members of breast cancer families, and matched healthy female controls.	plasma (ultracentrifugation)	nest-qPCR	For patients with early stage or localized breast cancer, there were high levels of miR-155 in both plasma and blood cells.	[98]
miR-130a-3p (↓)	40 pairs of breast cancer and adjacent normal tissues, 40 pairs of blood samples from patients with breast cancer and healthy controls (confirmed as invasive ductal breast cancer, and no patient had received any chemotherapy or radiotherapy ahead of surgery.)	circulating blood (ExoQuick Exosomal Extraction Kit)		Lower levels of exosome-derived miR-130a-3p are associated with lymph node metastasis (*p* = 0.0019) and advanced TNM stage (*p* = 0.0014).	[99]
miR-16 (↑), 30b (↓), 93 (↑)	111 BC patients, 42 DCIS patients and 39 healthy women	plasma	TaqMan real-time PCR	(1) The levels of exosomal miR-16 were higher in plasma of BC (*p* = 0.034) and DCIS (*p* = 0.047) patients than healthy women and were associated with estrogen (*p* = 0.004) and progesterone (*p* = 0.008) receptor status. (2) In estrogen-positive patients miR-16 was significantly enriched in exosomes (*p* = 0.0001). (3) Lower levels of exosomal miR-30b were associated with recurrence (*p* = 0.034). (4) Exosomal miR-93 was upregulated in DCIS patients (*p* = 0.001).	[100]
miR-200c (↑), -141 (↑)	259 human subjects, including 114 patients with breast cancer, 30 patients with benign breast tumors, 21 women with a family history of breast cancer, and 94 healthy women	plasma (ultracentrifugation)	nest-qPCR	Circulating levels of miR-200c and miR-141 are potential biomarkers for early detection of breast cancer metastases.	[101]
miR-223-3p (↑)	185 breast cancer patients, 20 healthy volunteers	plasma (ultracentrifugation)	microRNA (miRNA) microarray; RT-qPCR	(1) identify the invasive lesions of DCIS patients diagnosed by biopsy; (2) significantly associated with the malignancy of breast cancer.	[102]
miR-21(↑), 105(↑), 155(↑)	53 breast cancer women (6 of them were diagnosed as metastatic patients) and 8 healthy donors	serum	qPCR	During neoadjuvant treatment, exosomal miRNA-21 expression levels directly correlated with tumor size (*p* = 0.039) and inversely with Ki67 expression (*p* = 0.031).	[103]
miR-340-5p (↑), 17-5p (↓), 130a-3p (↓), 93-5p (↓)	16 patients with primary breast cancer with recurrence and 16 without recurrence; 35 breast cancer patients with and 39 without recurrence	serum (ExoQuick)	qRT-PCR	There are different expression patterns of miRNAs between tumor tissues and serum	[104]

**Table 5:** EVs as drug delivery agents for cancer therapy.

Therapeutic Cargo	EV Source	Recipient Cells	Target Gene	Drug Loading Techniques/POSSIBLE Drugs	Biological Activities	Key Findings	Refs
antagomiR-222/223	MSCs	Cancer cell (MDA-MB-231, T47D)		Co-transfection (Lipofectamine RNAiMAX Reagent)	cycle quiescence; dormancy; drug resistance (carboplatin)	A novel therapeutic strategy to target dormant breast cancer cells.	[85]
miR-130a, 425 (↓)	MCF-7		associated with the mTOR, ErbB, MAPK and TGF-β signaling pathways	DRβ-H	cell proliferation	DRβ-H inhibited MCF-7/S cell growth through reducing exosome release.	[114]
miR-128 (↓)	Cancer cell (MCF-7)	Cancer cell (MCF-7)	Bax	Shikonin (SK)	cell proliferation	shikonin inhibits the proliferation of MCF-7 cells by reducing tumor-derived exosomal miR-128.	[115]
miR-140 (↑)	Mouse preadipocyte (3T3L1, MBA-1)	MCF10DCIS cells	SOX9	Shikonin (SK)	tumorigenesis; regulating differentiation, stemness, and migration	(1) MiR-140/SOX2/SOX9 axis can regulate differentiation, stemness, and migration. (2) SK-treated preadipocytes secrete exosomes with high levels of miR-140, which can inhibit nearby DCIS cells by targeting SOX9 signaling	[116]
miR-16 (↑)	Cancer cell (4T1)	TAMs (RAW264.7)		EGCG	TME	EGCG up-regulates miR-16 in tumor cells, which can be transferred to TAM via exosomes and inhibits TAM infiltration and M2 polarization	[117]
miR-34a (↑), 452 (↓)	Cancer cell (MCF-7/Doc, MCF-7/Adr)	Cancer cell		β-elemene	reverse drug resistance (docetaxel, adriamycin)	β-elemene effectively sensitizes drug-resistant BCA cells to Doc and Adr through a signaling pathway that involves miRNA and gene regulation	[118]
miR-23b, 320b (↑)	Cancer cell (MDA-MB-231, MCF-7, ZR751 and BT20)	Epithelial cells (MCF-10A, EA.hy926)	PLAU, AMOTL1 (miR-23b); NRP1, ETS2 (miR-320b)	DHA	angiogenesis	the microRNAs transferred by exosomes mediate DHA’s anti-angiogenic action.	[119]
let-7a	Donor cells (express the transmembrane domain of PDGF fused to the GE11 peptide)	EGFR-expressing breast cancer cells		Modified exosomes with the GE11 peptide or EGF on their surfaces		(1) Modified exosomes with the GE11 peptide or EGF on their surfaces delivered miRNA to EGFR-expressing cancer tissues; (2) intravenously injected exosomes targeting EGFR delivered let-7a specifically to xenograft breast cancer cells in RAG2^−/−^mice.	[120]
miR-379 (↑)	Engineered MSCs	Cancer cells (T47D, HCC-1954)	COX-2	lentiviral transduction		Exploiting the tumor-homing capacity of MSCs while engineering the cells to secrete EVs enriched with miR-379 holds exciting potential as an innovative therapy for metastatic breast cancer.	[121]
miR-155, -142, and let-7i (↑)	Cancer cells (4T1)	Dendritic cells	IL-6, IL-17, IL-1b, TGF-β, SOCS1, KLRK1, IFN-γ, and TLR4	electroporation		The modified exosomes would be a hopeful cell-free vaccine for cancer treatment.	[122]
anti-miR-142-3p oligonucleotides	MSCs	Cancer cell (4T1 and TUBO)	APC (miR-142-3p); P2 × 7R (miR-150)	LNA (locked nucleic acid)-modified		MSCs-derived exosomes could be used as a feasible nano-vehicle to deliver drug molecules like LNA-anti-miR-142-3p in both in vitro and in vivo studies.	[125]

## References

[1] Cancer Genome Atlas N. (2012). Comprehensive molecular portraits of human breast tumours. Nature.

[2] Siegel R.L., Miller K.D., Jemal A. (2018). Cancer statistics. CA Cancer J. Clin..

[3] DeSantis C.E., Ma J., Goding Sauer A., Newman L.A., Jemal A. (2017). Breast cancer statistics, 2017, racial disparity in mortality by state. CA Cancer J. Clin..

[4] Aleskandarany M.A., Vandenberghe M.E., Marchio C., Ellis I.O., Sapino A., Rakha E.A. (2018). Tumour Heterogeneity of Breast Cancer: From Morphology to Personalised Medicine. Pathobiology.

[5] Curigliano G., Burstein H.J., P Winer E., Gnant M., Dubsky P., Loibl S., Colleoni M., Regan M.M., Piccart-Gebhart M., Senn H.J. (2017). De-escalating and escalating treatments for early-stage breast cancer: The St. Gallen International Expert Consensus Conference on the Primary Therapy of Early Breast Cancer. Ann. Oncol..

[6] Coates A.S., Winer E.P., Goldhirsch A., Gelber R.D., Gnant M., Piccart-Gebhart M., Thurlimann B., Senn H.J., Panel M. (2015). Tailoring therapies—Improving the management of early breast cancer: St Gallen International Expert Consensus on the Primary Therapy of Early Breast Cancer. Ann. Oncol..

[7] Kurozumi S., Yamaguchi Y., Kurosumi M., Ohira M., Matsumoto H., Horiguchi J. (2017). Recent trends in microRNA research into breast cancer with particular focus on the associations between microRNAs and intrinsic subtypes. J. Hum. Genet..

[8] Elston C.W., Ellis I.O., Pinder S.E. (1999). Pathological prognostic factors in breast cancer. Crit. Rev.Oncol. Hematol..

[9] Langley R.R., Fidler I.J. (2011). The seed and soil hypothesis revisited—The role of tumor-stroma interactions in metastasis to different organs. Int. J. Cancer.

[10] Ribatti D., Mangialardi G., Vacca A. (2006). Stephen Paget and the ‘seed and soil’ theory of metastatic dissemination. Clin. Exp. Med..

[11] Paget S.J.T.L. (1889). The distribution of secondary growths in cancer of the breast. Lancet.

[12] Soysal S.D., Tzankov A., Muenst S.E. (2015). Role of the Tumor Microenvironment in Breast Cancer. Pathobiology.

[13] Suzuki H.I., Katsura A., Matsuyama H., Miyazono K. (2015). MicroRNA regulons in tumor microenvironment. Oncogene.

[14] Hui L., Chen Y. (2015). Tumor microenvironment: Sanctuary of the devil. Cancer Lett..

[15] Kalluri R., Zeisberg M. (2006). Fibroblasts in cancer. Nat. Rev. Cancer.

[16] Kalluri R. (2016). The biology and function of fibroblasts in cancer. Nat. Rev. Cancer.

[17] Liao Z., Tan Z.W., Zhu P., Tan N.S. (2018). Cancer-associated fibroblasts in tumor microenvironment—Accomplices in tumor malignancy. Cell. Immunol..

[18] Shiga K., Hara M., Nagasaki T., Sato T., Takahashi H., Takeyama H. (2015). Cancer-Associated Fibroblasts: Their Characteristics and Their Roles in Tumor Growth. Cancers.

[19] Weber C.E., Kothari A.N., Wai P.Y., Li N.Y., Driver J., Zapf M.A., Franzen C.A., Gupta G.N., Osipo C., Zlobin A. (2015). Osteopontin mediates an MZF1-TGF-beta1-dependent transformation of mesenchymal stem cells into cancer-associated fibroblasts in breast cancer. Oncogene.

[20] McDonald L.T., LaRue A.C. (2012). Hematopoietic stem cell derived carcinoma-associated fibroblasts: A novel origin. Int. J. Clin. Exp. Pathol..

[21] Orimo A., Gupta P.B., Sgroi D.C., Arenzana-Seisdedos F., Delaunay T., Naeem R., Carey V.J., Richardson A.L., Weinberg R.A. (2005). Stromal fibroblasts present in invasive human breast carcinomas promote tumor growth and angiogenesis through elevated SDF-1/CXCL12 secretion. Cell.

[22] Nagasaki T., Hara M., Nakanishi H., Takahashi H., Sato M., Takeyama H. (2014). Interleukin-6 released by colon cancer-associated fibroblasts is critical for tumour angiogenesis: Anti-interleukin-6 receptor antibody suppressed angiogenesis and inhibited tumour-stroma interaction. Br. J. Cancer.

[23] Giulianelli S., Cerliani J.P., Lamb C.A., Fabris V.T., Bottino M.C., Gorostiaga M.A., Novaro V., Gongora A., Baldi A., Molinolo A. (2008). Carcinoma-associated fibroblasts activate progesterone receptors and induce hormone independent mammary tumor growth: A role for the FGF-2/FGFR-2 axis. Int. J. Cancer.

[24] Radisky E.S., Radisky D.C. (2007). Stromal induction of breast cancer: Inflammation and invasion. Rev. Endocr. Metab. Disord..

[25] Kohlhapp F.J., Mitra A.K., Lengyel E., Peter M.E. (2015). MicroRNAs as mediators and communicators between cancer cells and the tumor microenvironment. Oncogene.

[26] Balkwill F.R., Capasso M., Hagemann T. (2012). The tumor microenvironment at a glance. J. Cell Sci..

[27] Mantovani A., Marchesi F., Malesci A., Laghi L., Allavena P. (2017). Tumour-associated macrophages as treatment targets in oncology. Nat. Rev. Clin. Oncol..

[28] Noy R., Pollard J.W. (2014). Tumor-associated macrophages: From mechanisms to therapy. Immunity.

[29] Williams C.B., Yeh E.S., Soloff A.C. (2016). Tumor-associated macrophages: Unwitting accomplices in breast cancer malignancy. NPJ Breast Cancer.

[30] Lim B., Woodward W.A., Wang X., Reuben J.M., Ueno N.T. (2018). Inflammatory breast cancer biology: The tumour microenvironment is key. Nat. Rev. Cancer.

[31] Green T.M., Alpaugh M.L., Barsky S.H., Rappa G., Lorico A. (2015). Breast Cancer-Derived Extracellular Vesicles: Characterization and Contribution to the Metastatic Phenotype. BioMed Res. Int..

[32] Wang M., Yu F., Ding H., Wang Y., Li P., Wang K. (2019). Emerging Function and Clinical Values of Exosomal MicroRNAs in Cancer. Mol. Ther. Nucleic. Acids.

[33] Sempere L.F., Keto J., Fabbri M. (2017). Exosomal MicroRNAs in Breast Cancer towards Diagnostic and Therapeutic Applications. Cancers.

[34] Kharaziha P., Ceder S., Li Q., Panaretakis T. (2012). Tumor cell-derived exosomes: A message in a bottle. Biochim. Biophys. Acta.

[35] Hannafon B.N., Ding W.Q. (2013). Intercellular communication by exosome-derived microRNAs in cancer. Int. J. Mol. Sci..

[36] Xu R., Rai A., Chen M., Suwakulsiri W., Greening D.W., Simpson R.J. (2018). Extracellular vesicles in cancer—Implications for future improvements in cancer care. Nat. Rev. Clin. Oncol..

[37] Mulcahy L.A., Pink R.C., Carter D.R. (2014). Routes and mechanisms of extracellular vesicle uptake. J. Extracell. Vesicles.

[38] Meng Y., Sun J., Wang X., Hu T., Ma Y., Kong C., Piao H., Yu T., Zhang G. (2019). Exosomes: A Promising Avenue for the Diagnosis of Breast Cancer. Technol. Cancer Res. Treat..

[39] Bahrami A., Aledavood A., Anvari K., Hassanian S.M., Maftouh M., Yaghobzade A., Salarzaee O., ShahidSales S., Avan A. (2018). The prognostic and therapeutic application of microRNAs in breast cancer: Tissue and circulating microRNAs. J. Cell. Physiol..

[40] Record M. (2014). Intercellular communication by exosomes in placenta: A possible role in cell fusion?. Placenta.

[41] Teng X., Chen L., Chen W., Yang J., Yang Z., Shen Z. (2015). Mesenchymal Stem Cell-Derived Exosomes Improve the Microenvironment of Infarcted Myocardium Contributing to Angiogenesis and Anti-Inflammation. Cell. Physiol. Biochem..

[42] Greening D.W., Nguyen H.P., Elgass K., Simpson R.J., Salamonsen L.A. (2016). Human Endometrial Exosomes Contain Hormone-Specific Cargo Modulating Trophoblast Adhesive Capacity: Insights into Endometrial-Embryo Interactions. Biol. Reprod..

[43] Di Ieva A., Butz H., Niamah M., Rotondo F., De Rosa S., Sav A., Yousef G.M., Kovacs K., Cusimano M.D. (2014). MicroRNAs as biomarkers in pituitary tumors. Neurosurgery.

[44] Lee R.C., Feinbaum R.L., Ambros V.J. (1993). The C. elegans heterochronic gene lin-4 encodes small RNAs with antisense complementarity to lin-14. Cell.

[45] Berindan-Neagoe I., Monroig Pdel C., Pasculli B., Calin G.A. (2014). MicroRNAome genome: A treasure for cancer diagnosis and therapy. CA Cancer J. Clin..

[46] Li Z., Rana T.M. (2014). Therapeutic targeting of microRNAs: Current status and future challenges. Nat. Rev. Drug Discov..

[47] Challagundla K.B., Fanini F., Vannini I., Wise P., Murtadha M., Malinconico L., Cimmino A., Fabbri M. (2014). microRNAs in the tumor microenvironment: Solving the riddle for a better diagnostics. Expert Rev. Mol. Diagn..

[48] Zhang H., Li Y., Lai M. (2010). The microRNA network and tumor metastasis. Oncogene.

[49] Huang Q., Gumireddy K., Schrier M., le Sage C., Nagel R., Nair S., Egan D.A., Li A., Huang G., Klein-Szanto A.J. (2008). The microRNAs miR-373 and miR-520c promote tumour invasion and metastasis. Nat. Cell Biol..

[50] Zhong S., Chen X., Wang D., Zhang X., Shen H., Yang S., Lv M., Tang J., Zhao J. (2016). MicroRNA expression profiles of drug-resistance breast cancer cells and their exosomes. Oncotarget.

[51] Chen W.X., Xu L.Y., Qian Q., He X., Peng W.T., Zhu Y.L., Cheng L. (2018). Analysis of miRNA signature differentially expressed in exosomes from adriamycin-resistant and parental human breast cancer cells. Biosci. Rep..

[52] Guzman N., Agarwal K., Asthagiri D., Yu L., Saji M., Ringel M.D., Paulaitis M.E. (2015). Breast Cancer-Specific miR Signature Unique to Extracellular Vesicles Includes “microRNA-like” tRNA Fragments. Mol. Cancer Res. MCR.

[53] Kruger S., Abd Elmageed Z.Y., Hawke D.H., Worner P.M., Jansen D.A., Abdel-Mageed A.B., Alt E.U., Izadpanah R. (2014). Molecular characterization of exosome-like vesicles from breast cancer cells. BMC Cancer.

[54] Stevic I., Muller V., Weber K., Fasching P.A., Karn T., Marme F., Schem C., Stickeler E., Denkert C., van Mackelenbergh M. (2018). Specific microRNA signatures in exosomes of triple-negative and HER2-positive breast cancer patients undergoing neoadjuvant therapy within the GeparSixto trial. BMC Med..

[55] Fish E.J., Irizarry K.J., DeInnocentes P., Ellis C.J., Prasad N., Moss A.G., Curt Bird R. (2018). Malignant canine mammary epithelial cells shed exosomes containing differentially expressed microRNA that regulate oncogenic networks. BMC Cancer.

[56] Liu T., Zhang Q., Zhang J., Li C., Miao Y.R., Lei Q., Li Q., Guo A.Y. (2019). EVmiRNA: A database of miRNA profiling in extracellular vesicles. Nucleic Acids Res..

[57] Melo S.A., Sugimoto H., O’Connell J.T., Kato N., Villanueva A., Vidal A., Qiu L., Vitkin E., Perelman L.T., Melo C.A. (2014). Cancer exosomes perform cell-independent microRNA biogenesis and promote tumorigenesis. Cancer Cell.

[58] Kosaka N., Iguchi H., Hagiwara K., Yoshioka Y., Takeshita F., Ochiya T. (2013). Neutral sphingomyelinase 2 (nSMase2)-dependent exosomal transfer of angiogenic microRNAs regulate cancer cell metastasis. J. Biol. Chem..

[59] Singh R., Pochampally R., Watabe K., Lu Z., Mo Y.Y. (2014). Exosome-mediated transfer of miR-10b promotes cell invasion in breast cancer. Mol. Cancer.

[60] Ma L., Teruya-Feldstein J., Weinberg R.A. (2007). Tumour invasion and metastasis initiated by microRNA-10b in breast cancer. Nature.

[61] Li X.J., Ren Z.J., Tang J.H., Yu Q. (2017). Exosomal MicroRNA MiR-1246 Promotes Cell Proliferation, Invasion and Drug Resistance by Targeting CCNG2 in Breast Cancer. Cell. Physiol. Biochem..

[62] Wei Y., Lai X., Yu S., Chen S., Ma Y., Zhang Y., Li H., Zhu X., Yao L., Zhang J. (2014). Exosomal miR-221/222 enhances tamoxifen resistance in recipient ER-positive breast cancer cells. Breast Cancer Res. Treat..

[63] Yu D.D., Wu Y., Zhang X.H., Lv M.M., Chen W.X., Chen X., Yang S.J., Shen H., Zhong S.L., Tang J.H. (2016). Exosomes from adriamycin-resistant breast cancer cells transmit drug resistance partly by delivering miR-222. Tumour Biol..

[64] Chen W.X., Cai Y.Q., Lv M.M., Chen L., Zhong S.L., Ma T.F., Zhao J.H., Tang J.H. (2014). Exosomes from docetaxel-resistant breast cancer cells alter chemosensitivity by delivering microRNAs. Tumour Biol..

[65] Chen W.X., Liu X.M., Lv M.M., Chen L., Zhao J.H., Zhong S.L., Ji M.H., Hu Q., Luo Z., Wu J.Z. (2014). Exosomes from drug-resistant breast cancer cells transmit chemoresistance by a horizontal transfer of microRNAs. PLoS ONE.

[66] Mao L., Li J., Chen W.X., Cai Y.Q., Yu D.D., Zhong S.L., Zhao J.H., Zhou J.W., Tang J.H. (2016). Exosomes decrease sensitivity of breast cancer cells to adriamycin by delivering microRNAs. Tumour Biol..

[67] Kia V., Paryan M., Mortazavi Y., Biglari A., Mohammadi-Yeganeh S. (2019). Evaluation of exosomal miR-9 and miR-155 targeting PTEN and DUSP14 in highly metastatic breast cancer and their effect on low metastatic cells. J. Cell. Biochem..

[68] Kia V., Mortazavi Y., Paryan M., Biglari A., Mohammadi-Yeganeh S. (2019). Exosomal miRNAs from highly metastatic cells can induce metastasis in non-metastatic cells. Life Sci..

[69] Mihelich B.L., Dambal S., Lin S., Nonn L. (2016). miR-182, of the miR-183 cluster family, is packaged in exosomes and is detected in human exosomes from serum, breast cells and prostate cells. Oncol. Lett..

[70] O’Brien K., Lowry M.C., Corcoran C., Martinez V.G., Daly M., Rani S., Gallagher W.M., Radomski M.W., MacLeod R.A., O’Driscoll L. (2015). miR-134 in extracellular vesicles reduces triple-negative breast cancer aggression and increases drug sensitivity. Oncotarget.

[71] Zhou W., Fong M.Y., Min Y., Somlo G., Liu L., Palomares M.R., Yu Y., Chow A., O’Connor S.T., Chin A.R. (2014). Cancer-secreted miR-105 destroys vascular endothelial barriers to promote metastasis. Cancer Cell.

[72] Di Modica M., Regondi V., Sandri M., Iorio M.V., Zanetti A., Tagliabue E., Casalini P., Triulzi T. (2017). Breast cancer-secreted miR-939 downregulates VE-cadherin and destroys the barrier function of endothelial monolayers. Cancer Lett..

[73] Yan W., Wu X., Zhou W., Fong M.Y., Cao M., Liu J., Liu X., Chen C.H., Fadare O., Pizzo D.P. (2018). Cancer-cell-secreted exosomal miR-105 promotes tumour growth through the MYC-dependent metabolic reprogramming of stromal cells. Nat. Cell Biol..

[74] Fong M.Y., Zhou W., Liu L., Alontaga A.Y., Chandra M., Ashby J., Chow A., O’Connor S.T., Li S., Chin A.R. (2015). Breast-cancer-secreted miR-122 reprograms glucose metabolism in premetastatic niche to promote metastasis. Nat. Cell Biol..

[75] Hashimoto K., Ochi H., Sunamura S., Kosaka N., Mabuchi Y., Fukuda T., Yao K., Kanda H., Ae K., Okawa A. (2018). Cancer-secreted hsa-miR-940 induces an osteoblastic phenotype in the bone metastatic microenvironment via targeting ARHGAP1 and FAM134A. Proc. Natl. Acad. Sci. USA.

[76] Li Y., Liang Y., Sang Y., Song X., Zhang H., Liu Y., Jiang L., Yang Q. (2018). MiR-770 suppresses the chemo-resistance and metastasis of triple negative breast cancer via direct targeting of STMN. Cell Death Dis..

[77] Vaupel P., Mayer A. (2007). Hypoxia in cancer: Significance and impact on clinical outcome. Cancer Metastasis Rev..

[78] Jung K.O., Youn H., Lee C.H., Kang K.W., Chung J.K. (2017). Visualization of exosome-mediated miR-210 transfer from hypoxic tumor cells. Oncotarget.

[79] King H.W., Michael M.Z., Gleadle J.M. (2012). Hypoxic enhancement of exosome release by breast cancer cells. BMC Cancer.

[80] Baroni S., Romero-Cordoba S., Plantamura I., Dugo M., D’Ippolito E., Cataldo A., Cosentino G., Angeloni V., Rossini A., Daidone M.G. (2016). Exosome-mediated delivery of miR-9 induces cancer-associated fibroblast-like properties in human breast fibroblasts. Cell Death Dis..

[81] Dioufa N., Clark A.M., Ma B., Beckwitt C.H., Wells A. (2017). Bi-directional exosome-driven intercommunication between the hepatic niche and cancer cells. Mol. Cancer.

[82] Ono M., Kosaka N., Tominaga N., Yoshioka Y., Takeshita F., Takahashi R.U., Yoshida M., Tsuda H., Tamura K., Ochiya T. (2014). Exosomes from bone marrow mesenchymal stem cells contain a microRNA that promotes dormancy in metastatic breast cancer cells. Sci. Signal..

[83] Uen Y., Wang J.W., Wang C., Jhang Y., Chung J.Y., Tseng T., Sheu M., Lee S. (2018). Mining of potential microRNAs with clinical correlation—Regulation of syndecan-1 expression by miR-122-5p altered mobility of breast cancer cells and possible correlation with liver injury. Oncotarget.

[84] Santos J.C., Lima N.D.S., Sarian L.O., Matheu A., Ribeiro M.L., Derchain S.F.M. (2018). Exosome-mediated breast cancer chemoresistance via miR-155 transfer. Sci. Rep..

[85] Bliss S.A., Sinha G., Sandiford O.A., Williams L.M., Engelberth D.J., Guiro K., Isenalumhe L.L., Greco S.J., Ayer S., Bryan M. (2016). Mesenchymal Stem Cell-Derived Exosomes Stimulate Cycling Quiescence and Early Breast Cancer Dormancy in Bone Marrow. Cancer Res..

[86] Lim P.K., Bliss S.A., Patel S.A., Taborga M., Dave M.A., Gregory L.A., Greco S.J., Bryan M., Patel P.S., Rameshwar P. (2011). Gap junction-mediated import of microRNA from bone marrow stromal cells can elicit cell cycle quiescence in breast cancer cells. Cancer Res..

[87] Vallabhaneni K.C., Penfornis P., Dhule S., Guillonneau F., Adams K.V., Mo Y.Y., Xu R., Liu Y., Watabe K., Vemuri M.C. (2015). Extracellular vesicles from bone marrow mesenchymal stem/stromal cells transport tumor regulatory microRNA, proteins, and metabolites. Oncotarget.

[88] Deng Z., Rong Y., Teng Y., Zhuang X., Samykutty A., Mu J., Zhang L., Cao P., Yan J., Miller D. (2017). Exosomes miR-126a released from MDSC induced by DOX treatment promotes lung metastasis. Oncogene.

[89] Shah S.H., Miller P., Garcia-Contreras M., Ao Z., Machlin L., Issa E., El-Ashry D. (2015). Hierarchical paracrine interaction of breast cancer associated fibroblasts with cancer cells via hMAPK-microRNAs to drive ER-negative breast cancer phenotype. Cancer Biol. Ther..

[90] Donnarumma E., Fiore D., Nappa M., Roscigno G., Adamo A., Iaboni M., Russo V., Affinito A., Puoti I., Quintavalle C. (2017). Cancer-associated fibroblasts release exosomal microRNAs that dictate an aggressive phenotype in breast cancer. Oncotarget.

[91] Yang M., Chen J., Su F., Yu B., Su F., Lin L., Liu Y., Huang J.D., Song E. (2011). Microvesicles secreted by macrophages shuttle invasion-potentiating microRNAs into breast cancer cells. Mol. Cancer.

[92] Lee J.K., Park S.R., Jung B.K., Jeon Y.K., Lee Y.S., Kim M.K., Kim Y.G., Jang J.Y., Kim C.W. (2013). Exosomes derived from mesenchymal stem cells suppress angiogenesis by down-regulating VEGF expression in breast cancer cells. PLoS ONE.

[93] Pakravan K., Babashah S., Sadeghizadeh M., Mowla S.J., Mossahebi-Mohammadi M., Ataei F., Dana N., Javan M. (2017). MicroRNA-100 shuttled by mesenchymal stem cell-derived exosomes suppresses in vitro angiogenesis through modulating the mTOR/HIF-1alpha/VEGF signaling axis in breast cancer cells. Cell. Oncol..

[94] Bovy N., Blomme B., Freres P., Dederen S., Nivelles O., Lion M., Carnet O., Martial J.A., Noel A., Thiry M. (2015). Endothelial exosomes contribute to the antitumor response during breast cancer neoadjuvant chemotherapy via microRNA transfer. Oncotarget.

[95] Li M., Zhou Y., Xia T., Zhou X., Huang Z., Zhang H., Zhu W., Ding Q., Wang S. (2018). Circulating microRNAs from the miR-106a-363 cluster on chromosome X as novel diagnostic biomarkers for breast cancer. Breast Cancer Res. Treat..

[96] Hannafon B.N., Trigoso Y.D., Calloway C.L., Zhao Y.D., Lum D.H., Welm A.L., Zhao Z.J., Blick K.E., Dooley W.C., Ding W.Q. (2016). Plasma exosome microRNAs are indicative of breast cancer. Breast Cancer Res. BCR.

[97] Eichelser C., Stuckrath I., Muller V., Milde-Langosch K., Wikman H., Pantel K., Schwarzenbach H. (2014). Increased serum levels of circulating exosomal microRNA-373 in receptor-negative breast cancer patients. Oncotarget.

[98] Gao S., Wang Y., Wang M., Li Z., Zhao Z., Wang R.X., Wu R., Yuan Z., Cui R., Jiao K. (2017). MicroRNA-155, induced by FOXP3 through transcriptional repression of BRCA1, is associated with tumor initiation in human breast cancer. Oncotarget.

[99] Kong X., Zhang J., Li J., Shao J., Fang L. (2018). MiR-130a-3p inhibits migration and invasion by regulating RAB5B in human breast cancer stem cell-like cells. Biochem. Biophys. Res. Commun..

[100] Ni Q., Stevic I., Pan C., Muller V., Oliviera-Ferrer L., Pantel K., Schwarzenbach H. (2018). Different signatures of miR-16, miR-30b and miR-93 in exosomes from breast cancer and DCIS patients. Sci. Rep..

[101] Zhang G., Zhang W., Li B., Stringer-Reasor E., Chu C., Sun L., Bae S., Chen D., Wei S., Jiao K. (2017). MicroRNA-200c and microRNA-141 are regulated by a FOXP3-KAT2B axis and associated with tumor metastasis in breast cancer. Breast Cancer Res. BCR.

[102] Yoshikawa M., Iinuma H., Umemoto Y., Yanagisawa T., Matsumoto A., Jinno H. (2018). Exosome-encapsulated microRNA-223-3p as a minimally invasive biomarker for the early detection of invasive breast cancer. Oncol. Lett..

[103] Rodriguez-Martinez A., de Miguel-Perez D., Ortega F.G., Garcia-Puche J.L., Robles-Fernandez I., Exposito J., Martorell-Marugan J., Carmona-Saez P., Garrido-Navas M.D.C., Rolfo C. (2019). Exosomal miRNA profile as complementary tool in the diagnostic and prediction of treatment response in localized breast cancer under neoadjuvant chemotherapy. Breast Cancer Res. BCR.

[104] Sueta A., Yamamoto Y., Tomiguchi M., Takeshita T., Yamamoto-Ibusuki M., Iwase H. (2017). Differential expression of exosomal miRNAs between breast cancer patients with and without recurrence. Oncotarget.

[105] Zhao Q., Deng S., Wang G., Liu C., Meng L., Qiao S., Shen L., Zhang Y., Lu J., Li W. (2016). A direct quantification method for measuring plasma MicroRNAs identified potential biomarkers for detecting metastatic breast cancer. Oncotarget.

[106] Zhai L.Y., Li M.X., Pan W.L., Chen Y., Li M.M., Pang J.X., Zheng L., Chen J.X., Duan W.J. (2018). In Situ Detection of Plasma Exosomal MicroRNA-1246 for Breast Cancer Diagnostics by a Au Nanoflare Probe. ACS Appl. Mater. Interfaces.

[107] Zhang J., Wang L.L., Hou M.F., Xia Y.K., He W.H., Yan A., Weng Y.P., Zeng L.P., Chen J.H. (2018). A ratiometric electrochemical biosensor for the exosomal microRNAs detection based on bipedal DNA walkers propelled by locked nucleic acid modified toehold mediate strand displacement reaction. Biosens. Bioelectron..

[108] Sina A.A., Vaidyanathan R., Wuethrich A., Carrascosa L.G., Trau M. (2019). Label-free detection of exosomes using a surface plasmon resonance biosensor. Anal. Bioanal. Chem..

[109] Lee J.H., Kim J.A., Kwon M.H., Kang J.Y., Rhee W.J. (2015). In Situ single step detection of exosome microRNA using molecular beacon. Biomaterials.

[110] Lee J.H., Kim J.A., Jeong S., Rhee W.J. (2016). Simultaneous and multiplexed detection of exosome microRNAs using molecular beacons. Biosens. Bioelectron..

[111] Shao G., Ji S., Wu A., Liu C., Wang M., Zhang P., Jiao Q., Kang Y. (2015). DNAzyme-based probe for circulating microRNA detection in peripheral blood. Drug Des. Dev. Ther..

[112] Koumangoye R.B., Sakwe A.M., Goodwin J.S., Patel T., Ochieng J. (2011). Detachment of breast tumor cells induces rapid secretion of exosomes which subsequently mediate cellular adhesion and spreading. PLoS ONE.

[113] Manri C., Yokoi T., Nishida H. (2017). Size-Selective Harvesting of Extracellular Vesicles for Strategic Analyses Towards Tumor Diagnoses. Appl. Biochem. Biotechnol..

[114] Chen W.X., Cheng L., Pan M., Qian Q., Zhu Y.L., Xu L.Y., Ding Q. (2018). D Rhamnose beta-Hederin against human breast cancer by reducing tumor-derived exosomes. Oncol. Lett..

[115] Wei Y., Li M., Cui S., Wang D., Zhang C.Y., Zen K., Li L. (2016). Shikonin Inhibits the Proliferation of Human Breast Cancer Cells by Reducing Tumor-Derived Exosomes. Molecules.

[116] Gernapudi R., Yao Y., Zhang Y., Wolfson B., Roy S., Duru N., Eades G., Yang P., Zhou Q. (2015). Targeting exosomes from preadipocytes inhibits preadipocyte to cancer stem cell signaling in early-stage breast cancer. Breast Cancer Res. Treat..

[117] Jang J.Y., Lee J.K., Jeon Y.K., Kim C.W. (2013). Exosome derived from epigallocatechin gallate treated breast cancer cells suppresses tumor growth by inhibiting tumor-associated macrophage infiltration and M2 polarization. BMC Cancer.

[118] Zhang J., Zhang H.D., Yao Y.F., Zhong S.L., Zhao J.H., Tang J.H. (2015). beta-Elemene Reverses Chemoresistance of Breast Cancer Cells by Reducing Resistance Transmission via Exosomes. Cell. Physiol. Biochem..

[119] Hannafon B.N., Carpenter K.J., Berry W.L., Janknecht R., Dooley W.C., Ding W.Q. (2015). Exosome-mediated microRNA signaling from breast cancer cells is altered by the anti-angiogenesis agent docosahexaenoic acid (DHA). Mol. Cancer.

[120] Ohno S., Takanashi M., Sudo K., Ueda S., Ishikawa A., Matsuyama N., Fujita K., Mizutani T., Ohgi T., Ochiya T. (2013). Systemically injected exosomes targeted to EGFR deliver antitumor microRNA to breast cancer cells. Mol. Ther..

[121] O’Brien K.P., Khan S., Gilligan K.E., Zafar H., Lalor P., Glynn C., O’Flatharta C., Ingoldsby H., Dockery P., De Bhulbh A. (2018). Employing mesenchymal stem cells to support tumor-targeted delivery of extracellular vesicle (EV)-encapsulated microRNA-379. Oncogene.

[122] Taghikhani A., Hassan Z.M., Ebrahimi M., Moazzeni S.M. (2018). microRNA modified tumor-derived exosomes as novel tools for maturation of dendritic cells. J. Cell. Physiol..

[123] Lamichhane T.N., Jeyaram A., Patel D.B., Parajuli B., Livingston N.K., Arumugasaamy N., Schardt J.S., Jay S.M. (2016). Oncogene Knockdown via Active Loading of Small RNAs into Extracellular Vesicles by Sonication. Cell. Mol. Bioeng..

[124] Roma-Rodrigues C., Pereira F., Alves de Matos A.P., Fernandes M., Baptista P.V., Fernandes A.R. (2017). Smuggling gold nanoparticles across cell types—A new role for exosomes in gene silencing. Nanomed. Nanotechnol. Biol. Med..

[125] Naseri Z., Oskuee R.K., Jaafari M.R., Forouzandeh Moghadam M. (2018). Exosome-mediated delivery of functionally active miRNA-142-3p inhibitor reduces tumorigenicity of breast cancer in vitro and in vivo. Int. J. Nanomed..

